# Statistical Analysis of SSMIS Sea Ice Concentration Threshold at the Arctic Sea Ice Edge during Summer Based on MODIS and Ship-Based Observational Data

**DOI:** 10.3390/s18041109

**Published:** 2018-04-05

**Authors:** Qing Ji, Fei Li, Xiaoping Pang, Cong Luo

**Affiliations:** 1 Chinese Antarctic Center of Surveying and Mapping, Wuhan University, Wuhan 430079, China; jiqing@whu.edu.cn (Q.J.); pxp@whu.edu.cn (X.P.); 2 School of Resource and Environmental Sciences, Wuhan University, Wuhan 430079, China; luocong_18@hotmail.com

**Keywords:** sea ice concentration threshold, sea ice edge, passive microwave remote sensing, MODIS, ship-based observations

## Abstract

The threshold of sea ice concentration (SIC) is the basis for accurately calculating sea ice extent based on passive microwave (PM) remote sensing data. However, the PM SIC threshold at the sea ice edge used in previous studies and released sea ice products has not always been consistent. To explore the representable value of the PM SIC threshold corresponding on average to the position of the Arctic sea ice edge during summer in recent years, we extracted sea ice edge boundaries from the Moderate-resolution Imaging Spectroradiometer (MODIS) sea ice product (MOD29 with a spatial resolution of 1 km), MODIS images (250 m), and sea ice ship-based observation points (1 km) during the fifth (CHINARE-2012) and sixth (CHINARE-2014) Chinese National Arctic Research Expeditions, and made an overlay and comparison analysis with PM SIC derived from Special Sensor Microwave Imager Sounder (SSMIS, with a spatial resolution of 25 km) in the summer of 2012 and 2014. Results showed that the average SSMIS SIC threshold at the Arctic sea ice edge based on ice-water boundary lines extracted from MOD29 was 33%, which was higher than that of the commonly used 15% discriminant threshold. The average SIC threshold at sea ice edge based on ice-water boundary lines extracted by visual interpretation from four scenes of the MODIS image was 35% when compared to the average value of 36% from the MOD29 extracted ice edge pixels for the same days. The average SIC of 31% at the sea ice edge points extracted from ship-based observations also confirmed that choosing around 30% as the SIC threshold during summer is recommended for sea ice extent calculations based on SSMIS PM data. These results can provide a reference for further studying the variation of sea ice under the rapidly changing Arctic.

## 1. Introduction

In recent years, global climate change has drawn increasing attention [1]. Accelerated melting of the Arctic sea ice has led to a decline reflection of the Sun, allowing more solar radiation to be absorbed by the Arctic Ocean, further accelerating the ablation of the Arctic sea ice, and thus climate feedback. This “Arctic amplification” effect makes the Arctic Ocean a sensitive area influencing local and global climate changes [2].

Remote sensing is an important tool to monitor sea ice over a wide region. Passive microwave (PM) remote sensing can distinguish between sea ice and sea water, which are widely used to study the variation of sea ice extent at the Arctic scale [3,4,5,6]. Sea ice concentration (SIC) describes the relative amount of area covered by ice when compared to a satellite grid of a certain size [7]. However, a PM SIC threshold is needed to define whether a satellite cell is “ice-covered” or “open water”, to calculate the sea ice extent for trend analysis. For data cells with a SIC greater than or equal to the representative threshold value, the cell is regarded as an ice cell, while the others are excluded as open water.

A typical SIC threshold is 15%, which was first used by Parkinson et al. to define the location of the sea ice edge for measuring the Arctic sea ice extent [3]. According to the 15% SIC threshold, the sea ice extent in the northern hemisphere was analyzed over the period 1973–1987 using data derived from the Nimbus 5 Electrically Scanning Microwave Radiometer (ESMR) and the Nimbus 7 Scanning Multichannel Microwave Radiometer (SMMR) [8]. Cavalieri et al. followed the use of the 15% special sensor microwave imager (SSM/I) SIC threshold as the threshold of the location of sea ice edge for mapping Arctic sea ice [9]. However, Remund and Long compared the Ku band NSCAT scatterometer data with SSM/I radiometer derived SIC. They found that the NSCAT resulting edge closely matched the SSM/I derived 30% ice concentration edge [10]. Worby and Comiso also questioned the reliability of the sea-ice edge derived from 15% SIC after comparing Antarctic sea ice edge locations observed by SSM/I, ships, and a variety of remote sensing images [11].

In addition, the SIC threshold at the sea ice edge used in the released sea ice products is not always consistent. A threshold of 15% is used in the product of “Sea Ice Index” [12], and the product of “Sea Ice Trends and Climatologies from SMMR and SSM/I-SSMIS” [13]. However, some sea ice products used different SIC threshold values where a value of 20% is used in the product of “Arctic Sea Ice Freeboard and Thickness” [14], 30% is used in the product of “March through August Ice Edge Positions in the Nordic Seas” [15], and in the product of “CryoSat-2 Level 4 Sea Ice Elevation, Freeboard, and Thickness” [16], 35% is used in the product of “Global Products for Ice Concentration, Ice Edge, Ice Type, Sea Ice Drift” [17]. 

In these contexts, this paper focused on exploring a representative ice-water discrimination threshold for the SSMIS (with a spatial resolution of 25 km) in the Arctic in recent years. Section 2 describes the data used for comparing the SIC at the Arctic sea ice edge. Section 3 gives an overview of the sea ice edge extraction steps from satellite and ship-based sea ice observations. The comparison results are given in Section 4 and discussed in Section 5. The final section contains the conclusions drawn from the analysis.

## 2. Data

### 2.1. SSMIS Sea Ice Concentration

The sea ice concentration data [9] used in this paper for comparison at the Arctic sea ice edge were released by the National Snow and Ice Data Center (NSIDC), which was retrieved from the passive microwave sensor of SSMIS based on the NASS Team (NT) algorithm. The NT algorithm was first proposed by Cavalieri et al. and subsequently improved and updated by Gloersen and Cavalieri, and Cavalieri et al. [18,19,20], which has been widely used for SIC retrieval. The basic principle of the NT algorithm can be described as solving a set of linear algebraic equations where each equation decomposes the radiometric observation into components pertaining to the open water and ice types within the observation footprint, weighed by their concentration (Equation (1)) [21]:

TB_obs_ = C_ow_TB_ow_ + C_FY_TB_FY_ + C_MY_TB_MY_ (1)

where TB_obs_ is the observed brightness temperature; and TB_ow_, TB_FY_, TB_MY_ are the typical brightness temperatures from open water, first-year, and multi-year ice, respectively. C_ow_, C_FY_, C_MY_ are the concentrations of the three surfaces, respectively. In practice, the left hand side of Equation (1) are usually uses the polarization ratio (PR) and spectral gradient ratio (GR) both derived from brightness temperature, Equations (2) and (3) for the reason that they are independent of the physical temperature [21]:

PR = [TB(19V) ‒ TB(19H)]/[TB(19V) + TB(19H)](2)

GR = [TB(37V) ‒ TB(19V)]/[TB(37V) + TB(19V)](3)

where TB (f, p) is the satellite observed brightness temperature at the indicated frequency (f = 19 GHz or f = 37 GHz) and polarization (p = V or p = H). The measurement footprint size or effective field of view (FOV) of 19V, 19H and 37V used for the SIC NT algorithm are described in Table 1, and the spatial resolution were resampled by NSIDC to a pixel size of 25 km.

Based on the PR and GR, the first-year ice concentration (CF) and the multiyear ice concentration (CM) are calculated from the following equations:

CF = (a_0_ + a_1_PR + a_2_GR + a_3_PR × GR)/D (4)

CM = (b_0_ + b_1_PR + b_2_GR + b_3_PR × GR)/D (5)

D = c_0_ + c_1_PR + c_2_GR + c_3_PR × GR (6)

The total sea ice concentration (CT) is the sum of the first-year and multiyear concentrations:

CT = CF + CM(7)

The coefficients a_i_, b_i_, and c_i_ (i = 0,1,2,3) are functions of a set of brightness temperatures. These brightness temperatures, referred to as algorithm tie points, are the observed PM radiances over areas of known ice-free ocean, First Year (FY) sea ice, and Multi Year (MY) ice for each channel. The coefficients in Equations (3)–(5), are given in Table 2.

### 2.2. MOD29 Sea Ice Product

The MOD29 sea ice product is the MODIS daily sea ice product, which can be used to extract sea ice edges for a comparing with the SSMIS SIC. MOD29 data [22] used in this study were downloaded from the National Snow and Ice Data Center. The MOD29 sea ice product contains sea ice extent information for the HDF-EOS format, which also provides jpg format preview quick-look maps. The spatial resolution of the MOD29 data is 1 km, with the tiles grid based on a Lambert Azimuthal Equal-Area map projection. The MOD29 sea ice extent algorithm employs a grouped-criteria technique using the Normalized Difference Snow Index (NDSI) and other spectral threshold tests to identify snow and sea ice on a pixel-by-pixel basis [23]:

NDSI = (Ref_4_ − Ref_6_)/(Ref_4_ + Ref_6_) (8)

where Ref_4_ and Ref_6_ are the reflectance from MODIS bands 4 and 6, respectively. Then, the sea ice can be discriminated using the following satisfied criteria [22]:

(NDSI > 0.4) ∩ (Ref_2_ > 0.11) ∩ (Ref_4_ > 0.1) (9)

where Ref_2_ is the reflectance from MODIS band 2. With the MODIS land mask and cloud mask defined areas input, MOD29 data provide sea ice, ocean, land, and cloud information in the tile grid. 

### 2.3. MODIS Remote Sensing Images

The optical remote sensing images from MODIS sensor were used to make a visual interpretation of sea ice edges for comparing SSMIS SIC. The data were available from the Goddard Space Center website [24]. The MODIS sensor can provide images of a total of 36 separated spectral bands ranging in wavelength from 0.4–14.5 μm with a viewing swath width of 2330 km. The real-time surface conditions of sea ice and sea water were clearly shown and documented in [25]. In this study, the band-2 data in the MODIS L1B data set with 250 m resolution were chosen in the sea ice edge interpretation and comparison process. 

### 2.4. CHINARE-2012 and CHINARE-2014 Sea Ice Observations

**Figure 1:**
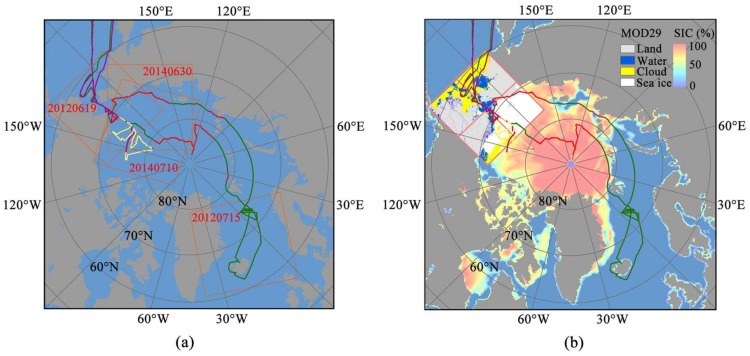
The R/V Xuelong cruise tracks of the fifth (CHINARE-2012) and the sixth (CHINARE-2014) Chinese National Arctic Research Expeditions, the green line represents CHINARE-2012 and the purple line CHINARE-2014, while the red and yellow points are the sea ice observations during CHINARE-2012 and CHINARE-2014, with the coverage of four MODIS images (orange rectangles within the labeled acquisition date) used in this study in (**a**); and the MOD29 data corresponding to the MODIS image on 19 June 2012 (pink rectangles) overlapping SSMIC SIC at the same day in (**b**).

The CHINARE-2012 and CHINARE-2014 were carried out aboard the R/V Xuelong from China between July and September in 2012 and 2014. One of the important scientific objectives of the expeditions was to explore the variation of Arctic sea ice. In this study, sea ice observations based on Xuelong during CHINARE-2012 and CHINARE-2014 [26] were used as a comparison with SSMIS SIC at the Arctic sea ice edge. The sea ice conditions along the ship tracks (Figure 1) were observed according to the Arctic Ship-based Sea Ice Standardization Tool (ASSIST) protocol, which is similar to the ASPeCt protocol for the Antarctic, and accepted as an international standard for sea ice visual observation [27]. According to the protocol, sea ice conditions including the ship’s position, sea ice concentration, sea ice type, and other ice variables are typically recorded hourly within a radius of 1 km around the ship.

## 3. Methods

### 3.1. Sea Ice Edge Lines Extraction from MOD29

To match the CHINARE-2014 and CHINARE-2014 sea ice ship-based observations, we selected MOD29 sea ice data from June to August in 2012 and 2014. According to the quick-look maps of the MOD29 product, a total of 69 MOD29 data with clear sea ice edges were finally selected for a comparison with the PM SIC, distributed at the Arctic marginal sea of the Chukchi Sea, the East Siberian Sea, the Laptev Sea, and the Greenland Sea (Table 3). 

**Figure 2:**
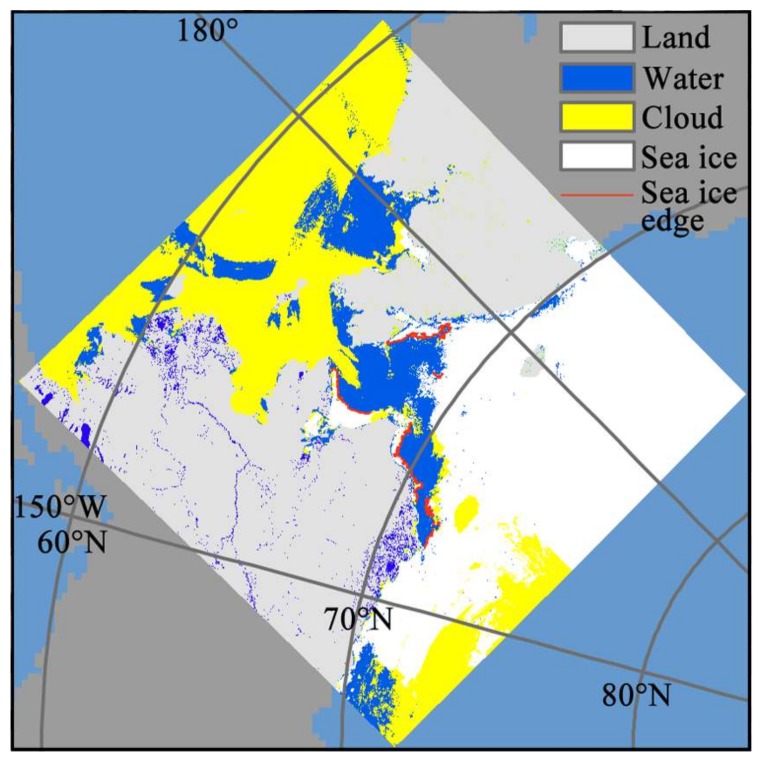
Sea ice edge lines extraction of the Chukchi Sea on 29 June 2012 from the MOD29 data.

As shown in Figure 2 (taking MOD29 data on 19 June 2012 in Chukchi Sea as an example), the grey color represents the land, while the yellow represents the cloud, and the intersections between the blue (seawater) and white part (sea ice) are the sea ice edges. Finally, we extracted the intersection lines (red lines in Figure 2) between the sea ice and seawater, and masked all the PM SIC pixels that the intersection lines passed through. The PM SIC at the Arctic sea ice edges were then calculated by the number of pixels, the average value, and the standard deviation. 

### 3.2. Sea Ice Edge Lines Visual Interpretation from MODIS Images

Aside from the extracted MOD29 sea ice edges, four senses MODIS images including 19 June 2012 of the Chukchi Sea, 15 July 2012 of the Greenland Seas, 30 June 2014 of the Laptev Sea, and 10 July 2014 of the East Siberian Sea with less cloud were selected for making the visual interpretation to extract sea ice edges.

**Figure 3:**
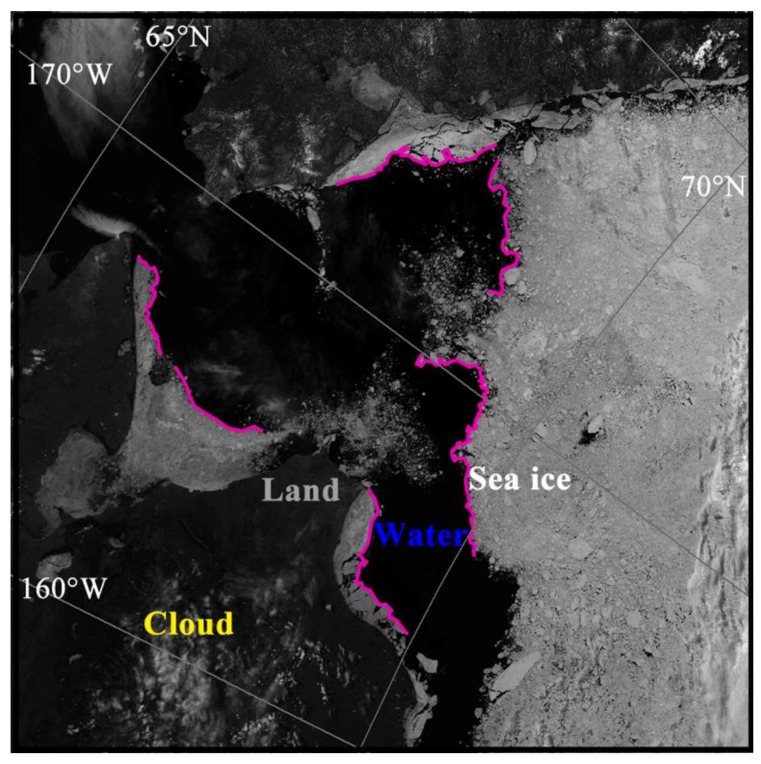
Sea ice edge lines (purple lines) visual interpretation of the Chukchi Sea on 29 June 2012 from the MODIS image.

Sea ice, land, cloud and seawater could be visually distinguished according to the grayscale and texture of the MODIS images [23]. Seawater had the minimum gray value, close to black, while sea ice had the maximum gray value, which seems partial to white, while the gray value of the land was between the two. The reticular light white part with linear texture was the cloud, whereas, sea ice had a grainy texture. Visual interpretation results of the sea ice edges (purple lines) are shown in Figure 3, using the image from 19 June 2012 of the Chukchi Sea as an example. We then extracted the intersection lines between the sea ice and seawater, and made the statistics of the PM SIC according to these extracted lines.

### 3.3. Sea Ice Edge Points Extraction from Ship-Based Observations

Sea ice edge points were also extracted from the CHINARE-2012 and CHINARE-2014 ship-based observations in this study. The extracting processing is described as follows.

**Figure 4:**
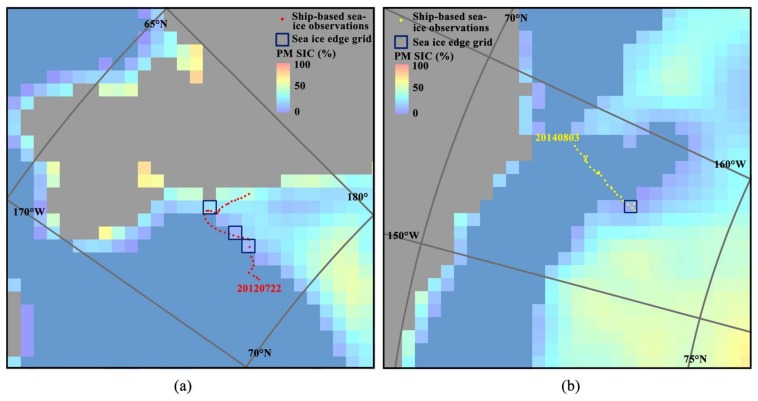
SSMIS SIC and the location of sea ice edge points observed by ship: (**a**) 22 July 2012 during CHINARE-2012 and (**b**) 3 August 2014 during CHINARE-2014.

First, the ship-based observation points were sorted by date. We then overlaid the SSMIS SIC data according to the same data, and extracted sea ice edge points where the ship-based observations points were adjacent to the sea water grid in the PM SIC grid. Finally, the number of sea ice edge points, the mean values from both ship-based observations, and the SSMIS SIC were calculated and compared. The example results (22 July 2012 and 3 August 2014) are shown in Figure 4.

### 3.4. SSMIS SIC Threshold Calculated Based on Extracted Sea Ice Edge Lines and Points

When the sea ice edge lines and points were extracted from relatively high resolution data such as MOD29 sea ice products, MODIS images, and ship-based observations, they were then overlaid to coincide with SSMIS SIC (with a spatial resolution of 25 km), in order to calculate the mean SIC at sea ice edges, which was used as the ice-water discrimination threshold for PM data. These comparisons were to explore the representative ice-water discrimination threshold for SSMIS in the Arctic in recent years.

## 4. Results

### 4.1. SSMIS SIC Threshold Based on MOD29 Sea Ice Edge Lines

**Figure 5:**
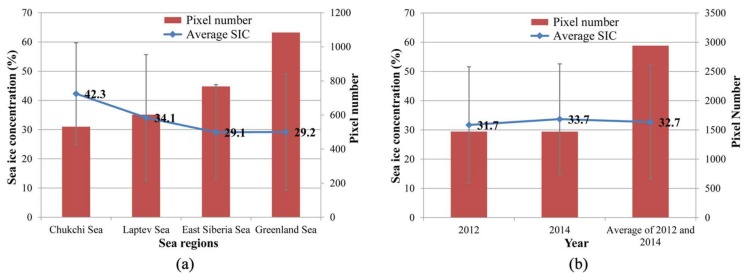
SSMIS SIC at the sea ice edge extracted from the MOD29 product for: (**a**) different sea regions; and (**b**) different years.

We calculated the SSMIS SIC corresponding to the extracted MOD29 sea ice edges in four different marginal seas. The results are shown in Figure 5a, where the highest SIC mean threshold value at the sea ice edge was the Chukchi Sea, which was 42%, followed by the Laptev Sea (34%), while the East-Siberian Sea and the Greenland Sea SIC threshold were both found to be 29%. 

From the statistical results of the different years (Figure 5b), we can see that the SSMIS SIC threshold at the Arctic sea ice edges in 2012 and 2014 were basically the same, with the value of 32% and 34%, respectively, and an average value of 33%.

### 4.2. SSMIS SIC Threshold Based on MODIS Sea Ice Edge Lines

The results of the SSMIS SIC at the Arctic sea ice edges corresponding to the visual interpretation from 4 senses MODIS images are shown in Table 4. It can be seen that the average SIC threshold at the Laptev Sea ice edge was the highest, reaching 37%, while the East Siberia Sea and the Greenland Sea were 34%, and the value of the Chukchi Sea was 33%. These threshold values were all higher than the commonly used threshold of 15% at the Arctic sea ice edge for time-series sea ice extent calculation.

In addition, by comparing the results from the MODIS images and from the corresponding MOD29 product, we found that the threshold values from MOD29 (36% on average) were generally higher than those from the MODIS images (35% on average).

### 4.3. SSMIS SIC Threshold Based on Ship-Bbserved Sea Ice Edge Points 

It can be seen from Table 5 that the average SIC at the Arctic sea ice edges of the ship observations was 31%, which was slightly higher than that of the corresponding SSMIS SIC (27%). Meanwhile, the correlation coefficient between the SIC of the ship-observed sea ice edge points and the corresponding SSMIS was 0.543, which shows the moderate correlation. 

## 5. Discussion

Currently, much attention has been paid to the variation and the trend of Arctic sea ice using passive microwave remotely sensed data [28,29], but studies on sea ice concentration thresholds based on these data were reported in a few cases [10,30]. The objective of this study was to explore the representable value of the PM SIC threshold corresponded on average to the position of the Arctic sea ice edge during summer in recent years. For this connection, the MODIS sea ice product (MOD29), MODIS images, as well as ship observational data from different Arctic marginal sea regions and different years were used to extract Arctic sea ice edges, and conduct an overlay analysis of the responding PM sea ice concentration threshold in the context of a rapid changing Arctic.

Overall, the thresholds obtained from the comparisons were rather similar; different comparison results indicated that the representable threshold value of Arctic sea ice edge during the summer was higher than the well-known 15% (Figure 5, Table 4 and Table 5). The mean threshold of 30% calculated in this study was consistent with that in [30], which compared NSCAT scatterometer data with radiometer derived SIC. The higher threshold value can be attributed to the following reasons. First, during the summer season, PM SIC at the ice edge with course resolution may rise from 0% at one pixel to 100% at its neighboring pixel, which result in mean SIC at the ice boundaries extracted from MOD29, MODIS images, and ship-based observational data were high at 30%, even at 40% due to the mixed pixel problem, especially for the declining and thinning Arctic sea ice in recent years. Second, the image of MODIS was a snapshot of the day, and the ship-based observations were at an hourly time-scale, however, the PM SIC was the daily average product. This may have caused the mismatch of the sea ice edges, especially on windy days when the sea ice edge moves at a high speed.

**Figure 6:**
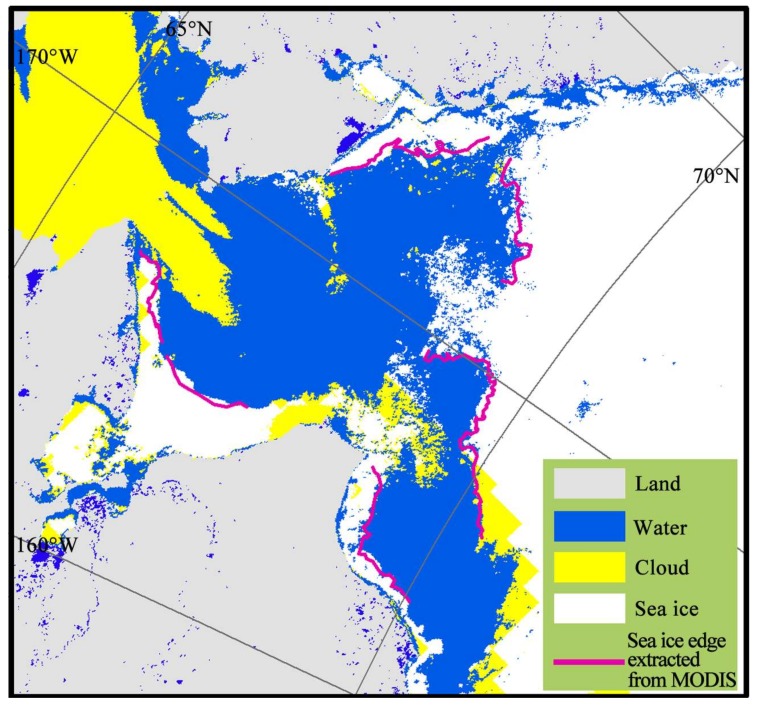
Comparison of sea ice edges extracted from MODIS and MOD29 sea ice product.

Although the threshold values from the MOD29 and MODIS images were both higher than 15%, MOD29 (36% on average) was generally higher than that from the corresponding MODIS images (35% on average). Figure 6 is the MOD29 sea ice product overlapping the sea ice edges interpretation from the MODIS image. From the purple line, which is the sea ice edge visual interpretation from the MODIS image, and the white, the blue, and the yellow parts which present the sea ice, seawater, and cloud from the MOD29 data, respectively, we can conclude that the MOD29 sea ice product would misjudge part of the seawater as sea ice, which results in a higher sea ice concentration at the sea ice edge corresponding to that extracted from the MODIS image. 

The correlation between the SIC of the ship-observed sea ice edge points and the corresponding SSMIS was moderate, which may be due to the following factors. First, there were some errors in the SSMIS SIC due to the SIC retrieval algorithm, as well as the existing errors for the sea ice ship-based observations due to the influence of in-situ human observations. Second, the spatial resolution between the SSMIS and the ship-based observation SIC was different as the resolution of the SSMIS data was 25 km and the ship observational data were 1 km.

It should be noted that different SIC algorithms, with the spatial resolution of each channel that enters into these SIC algorithms may influence the threshold results. Although Comiso and Parkinson [31], Heygster et al. [32], and Beitsch et al. [33] all reported that there were not significant biases of derived SIC among the different algorithms and sensors, however, these would be of great value to study in depth. We made the statistical analysis of SSMIS SIC based on the Bootstrap algorithm [34] and the ASI algorithm [35] at the ice-water boundary lines from a visual interpretation of the MODIS images. Comparing the results in Table 4 and Table 6, we found that the different SIC algorithms and different spatial resolution (e.g., 6.25 km for the ASI algorithm using a high-frequency channel) had some influence. However, the representable values of the PM SIC thresholds corresponding on average to the position of the Arctic sea ice edge were rather similar, which were all higher than the commonly used threshold of 15%. 

**Figure 7:**
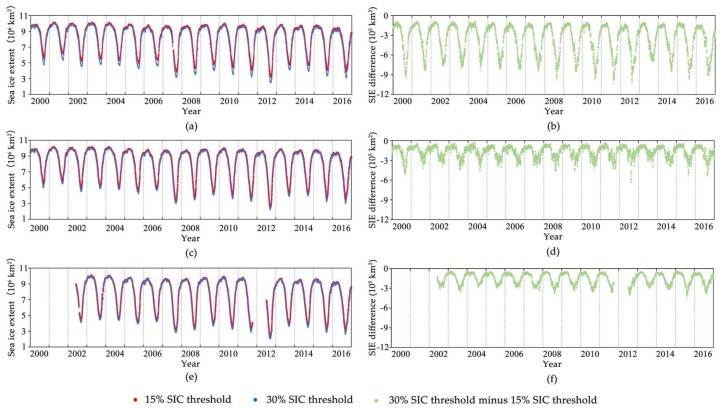
Influence of different SIC thresholds on the Arctic sea ice extent (SIE): (**a**,**c**,**e**) are the calculated SIE based on the NT SIC algorithm (25 km SSM/I and SSMIS PM data), Bootstrap SIC algorithm (25 km SSM/I and SSMIS PM data), and ASI SIC algorithm (6.25 km AMSR-E and AMSR2 PM data), respectively; (**b**,**d**,**f**) are their corresponding SIE differences between using a 30% SIC threshold and using a 15% SIC threshold.

Since a representable SIC threshold of 15% or 30% would influence the exact Arctic sea ice extent, we compared the difference of the calculated Arctic sea ice extent based on a 15% and 30% SIC threshold for both the NT SIC algorithm and Bootstrap SIC algorithm with a spatial resolution of 25 km for SSM/I and SSMIS data, and the ASI SIC algorithm with a spatial resolution of 6.25 km for AMSR-E and AMSR2 PM data. Figure 7 shows that there was a sea ice extent difference when using a 15% SIC threshold and 30% SIC threshold, especially during the summer season. 

The maximum differences of the calculated sea ice extent based on 15% and 30% SIC thresholds were 10.33 × 10^5^ km^2^ and 6.24 × 10^5^ km^2^ for the NT and Bootstrap SIC algorithms, and 3.99 × 10^5^ km^2^ for the ASI SIC algorithm with a spatial resolution of 6.25 km. The averaged relative differences at the minimum and maximum extents from 2002 to 2016 based on 15% and 30% SIC thresholds were 5.63 × 10^5^ km^2^ and 1.24 × 10^5^ km^2^ for the NT and Bootstrap SIC algorithms, and 1.48 × 10^5^ km^2^ for the ASI SIC algorithm. We deduce that the improvement of the SIC algorithms and the satellite sensors’ spatial resolution will reduce the impact of choosing different SIC thresholds on the calculated sea ice extent. However, these influences should not be ignored when using different SIC products for the long-term trend of Arctic sea ice extent.

## 6. Conclusions

This paper explored the representable value of the SSMIS sea ice concentration threshold at the Arctic sea ice edge during summer in recent years. The MOD29 sea ice product, MODIS images, and ship-based sea ice observations from CHINARE-2012 and CHINARE-2014 were used to extract Arctic sea ice edges. Based on these extracted ice edge lines and points, we made a comparison and statistical analysis of the SSMIS sea ice concentration at the Arctic sea ice edge in the summer of 2012 and 2014. 

Overlay analysis of the sea ice edges extracted from the MOD29 sea ice product and SSMIS SIC showed that the average SIC threshold of the four Arctic marginal seas was 33%. The average SIC threshold (42%) of the Chukchi Sea was higher than that in other marginal seas, 34% for the Laptev Sea, 29% for the East Siberian Sea, and 29% for the Greenland Sea. The average SIC thresholds in 2012 and 2014 were 32% and 34%, respectively, with an average value of 33%. Therefore, the commonly used 15% SIC threshold at the Arctic sea ice edge could be adjusted to 30% in the recently rapid changing Arctic.

A comparison of four scenes of MODIS visual interpretation sea ice edges and the corresponding SSMIS SIC indicated that the average SIC threshold value was 33% on 9 June 2012 for the Chukchi Sea, 37% for the Greenland Sea ice on 15 July 2012, 34% for the Laptev Sea on 30 June 2014, and 34% for the East Siberia Sea on 10 July 2014. All these values further confirmed that using 30% as the sea ice edge threshold with respect to the 15% was more reasonable.

The threshold SIC values from overlying the MOD29 and MODIS visual interpretation of the sea ice edge lines on the same date and the same sea region were approximately similar. MOD29 was slightly higher than that of the MODIS visual interpretation as the MOD29 sea ice product misjudged sea water as sea ice in some areas, resulting in a higher SIC at the sea ice edge.

The average SIC at the Arctic sea ice edge points extracted from ship-based observations during CHINARE-2012 and CHINARE-2014 was 31%, which also showed that choosing around 30% as the SIC threshold is recommended for sea ice extent calculation based on SSMIS SIC data. These results and conclusions can provide a reference for further development and improvement of PM SIC products and studying the variation of sea ice under the rapidly changing Arctic.

## Figures and Tables

**Table 1:** Effective field of view (FOV) for SSMIS channels and their resampled grid size.

Channel	FOV (km)	NSIDC Polar Stereographic Projection Grid Size (km)
19V	70 × 45	25
19H	70 × 45	25
37V	38 × 30	25

**Table 2:** Coefficients derived from tie points.

Coefficient	Value
a_0_	3290.2
a_1_	–20,761.2
a_2_	23,934.0
a_3_	47,985.4
b_0_	–790.9
b_1_	13,825.3
b_2_	–33,155.8
b_3_	–47,771.9
c_0_	2035.3
c_1_	9244.6
c_2_	–5665.8
c_3_	–12,875.1

**Table 3:** Number of selected MOD29 data with clear sea ice boundaries in different Arctic sea regions during summer in 2012 and 2014.

Arctic Sea Regions	2012			2014		
	June	July	August	June	July	August
Chukchi Sea	7	4	0	1	1	0
East Siberian Sea	0	1	1	4	4	8
Laptev Sea	2	3	0	6	3	1
Greenland Sea	5	7	4	3	2	2

**Table 4:** Statistics of SSMIS SIC at sea ice edges from MODIS images and from MOD29 products.

Date	Sea Regions	MODIS Visual Interpretation		MOD29 Extraction	
		Average (%)	Standard Deviation (%)	Average (%)	Standard Deviation (%)
19 June 2012	Chukchi Sea	33	14	36	13
15 July 2012	Greenland Sea	34	15	35	17
30 June 2014	Laptev Sea	37	17	38	21
10 July 2014	East Siberia Sea	34	17	35	7

**Table 5:** Statistics of SIC at sea ice edge observed points and their corresponding SSMIS SIC during CHINARE-2014 and CHINARE-2014.

Data	Ship-Observed SIC at Sea Ice Edge Points (%)	SSIMS SIC Corresponding to Ship-Observed Sea Ice Edge Points (%)
20 July 2012	50	43
22 July 2012	12	21
26 July 2012	35	28
27 July 2012	43	26
28 July 2012	31	23
4 September 2012	41	28
31 July 2014	17	31
2 August 2014	33	28
3 August 2014	39	21
31 August 2014	21	21
1 September 2014	15	22
Average	31	27

**Table 6:** Statistics of SSMIS SIC with a 25 km spatial resolution based on the Bootstrap algorithm and SSMIS SIC with a 6.25 km spatial resolution based on the ASI algorithm at the ice-water boundary lines from visual interpretation of the MODIS images.

Date	Sea Regions	SIC Based on Bootstrap Algorithm		SIC Based on ASI Algorithm	
		Average (%)	Standard Deviation (%)	Average (%)	Standard Deviation (%)
19 June 2012	Chukchi Sea	31	18	28	18
15 July 2012	Greenland Sea	29	16	32	16
30 June 2014	Laptev Sea	38	16	33	17
10 July 2014	East Siberia Sea	34	17	25	18
